# Supplementary material for: Template and target-site recognition by human LINE-1 in retrotransposition
Source: Nature. 2023 Dec 14;626(7997):186–93. doi: 10.1038/s41586-023-06933-5 (PMC10830416; doi:10.1038/s41586-023-06933-5)
Supplement: Supplementary file 1 — Supplementary Fig. 1 (uncropped gels for Figs. 1c,d, 2c,g, 3d,f and 4b–e and Extended Data Figs. 1a,c,d, 5e, 7a,b, 8 and 10b) and Supplementary Table 1 (all of the nucleic acid sequences used in this study). [file 41586_2023_6933_MOESM1_ESM.pdf]

nature portfolio

https://doi.org/10.1038/s41586-023-06933-5

Supplementary information

Template and target-site recognition by human LINE-1 in retrotransposition

In the format provided by the authors and unedited

Supplementary Figure 1

Figure 1c

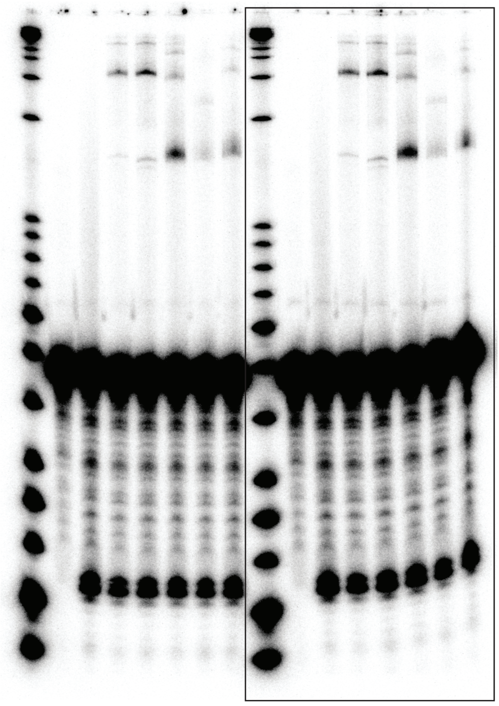

Figure 1d

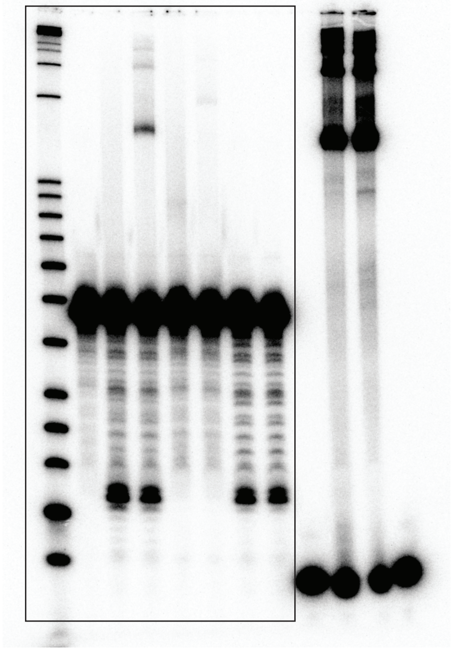

Figure 2c

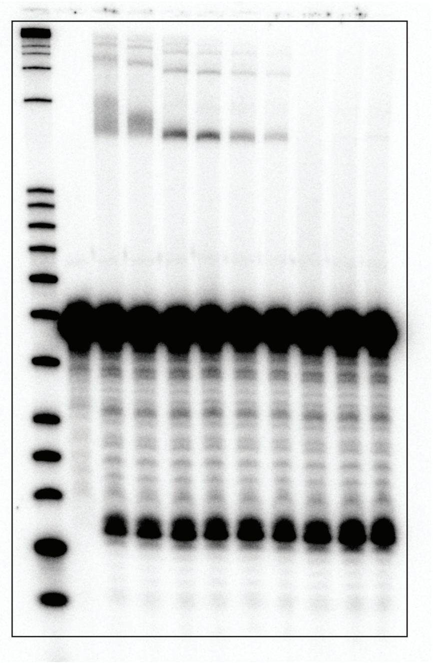

Figure 3d

Figure 2g

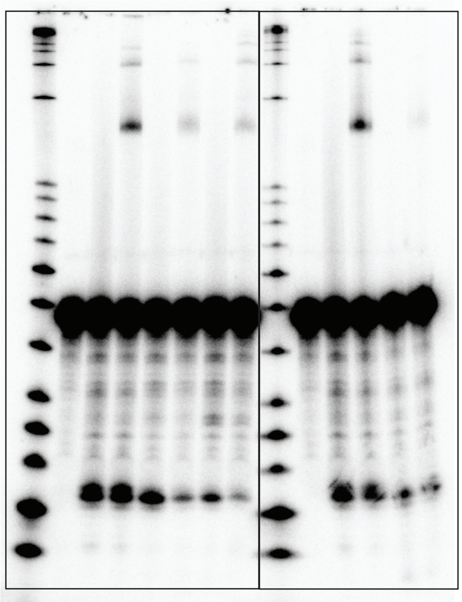

Figure 3f

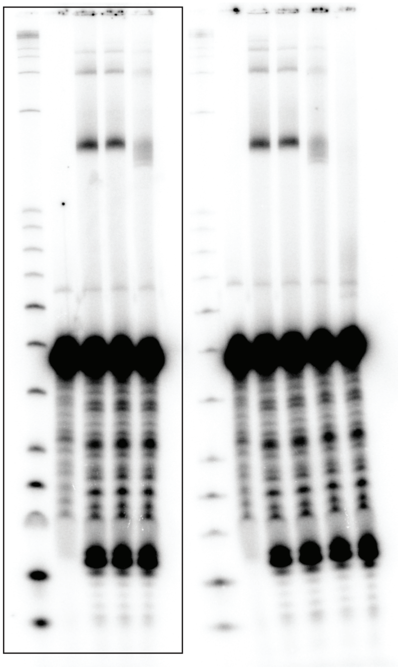

Figure 4b, Extended Data Fig 7a

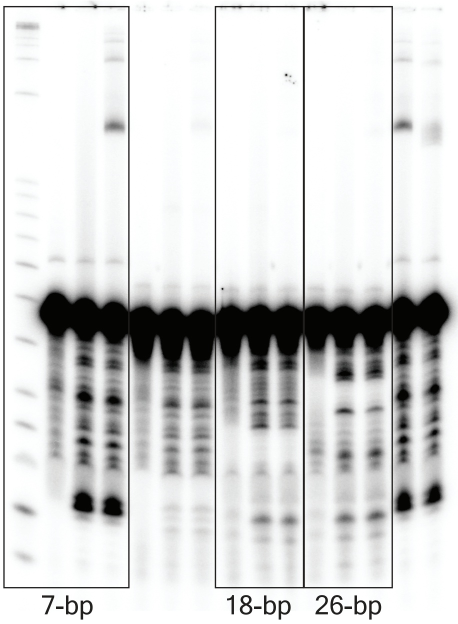

Supplementary Figure 1 (contd)

Figure 4c, , Extended Data Fig 7b

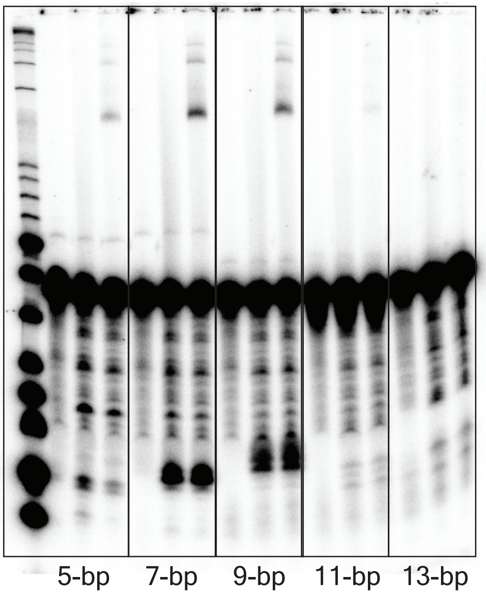

Figure 4d

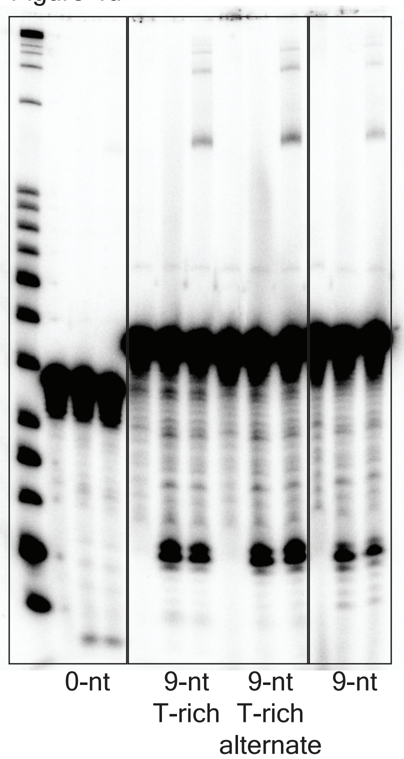

Figure 4e

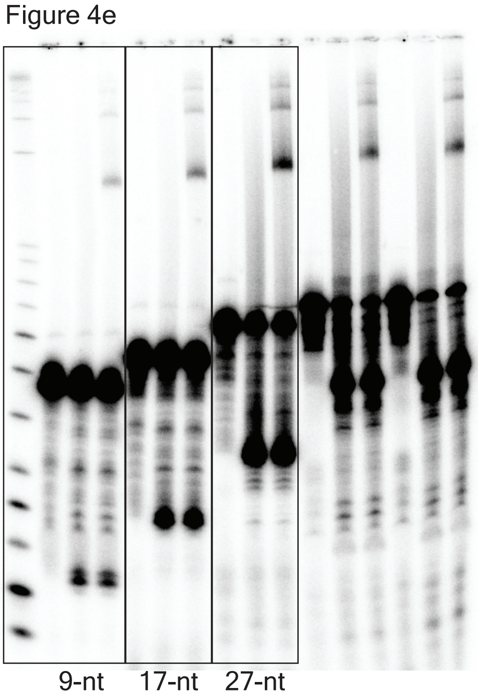

Extended Data Fig 1a

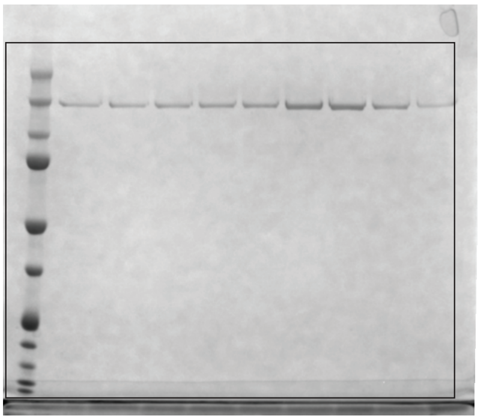

Extended Data Fig 1c

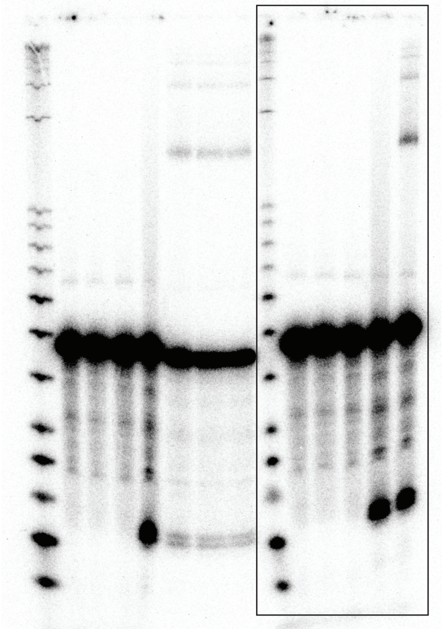

Supplementary Figure 1 (contd)

Extended Data Fig 1d

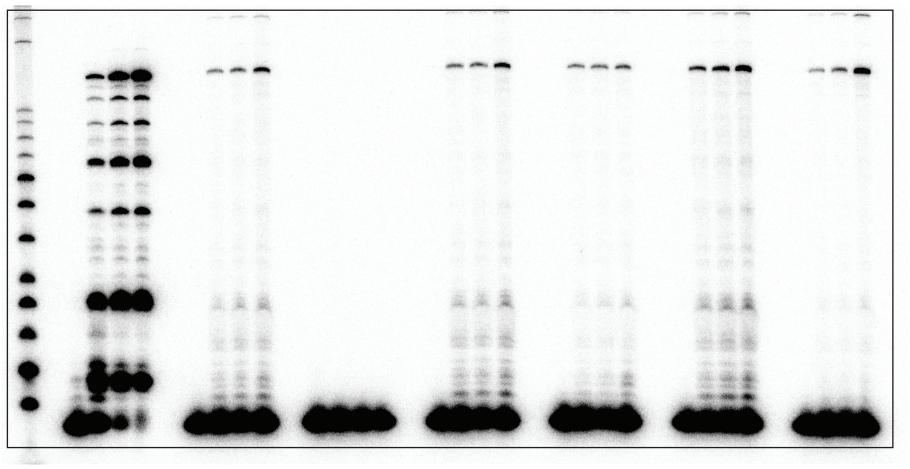

Extended Data Fig 5e

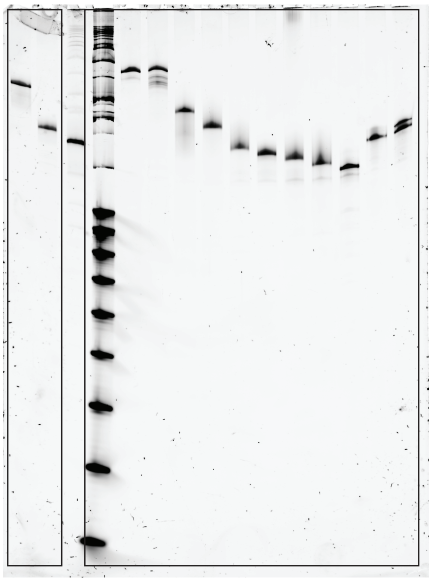

Extended Data Fig 8

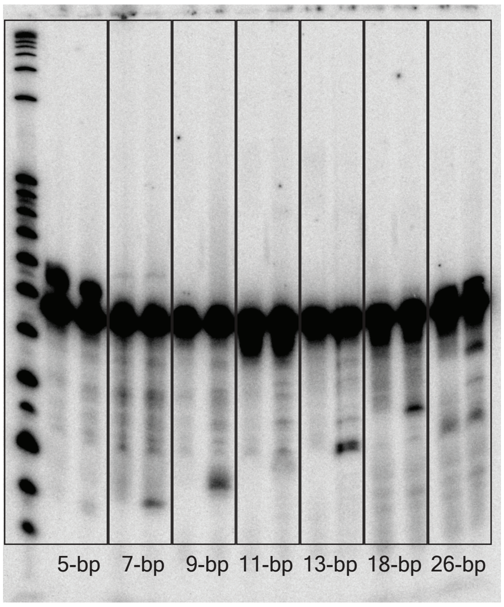

Extended Data Fig 10b

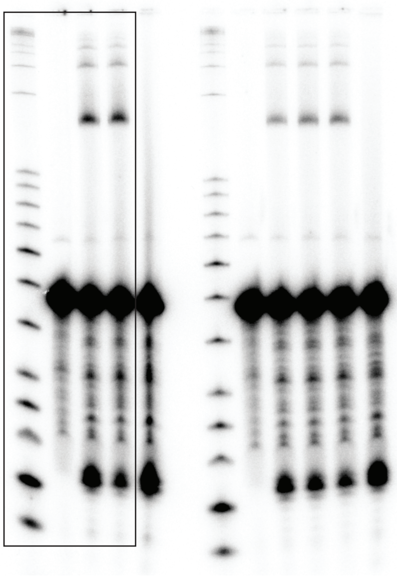

Supplementary Table 1. Nucleic acids used in this study

**Name**	**Sequence**	**Purpose and figure**
**RNA**		
AluY SINE 25A (AY, 307nt)	GACCGGCCGGGCGCGGUGGCUCACGCCUGUAA<br>UCCCAGCACUUUGGGAGGCCGAGGCGGGCGGA<br>UCACGAGGUCAGGAGAUCGAGACCAUCCCGGC<br>UAAAACGGUGAAACCCCGUCUCUACUAAAAAU<br>ACAAAAAAUUAGCCGGGCGUAGUGGCGGGCGC<br>CUGUAGUCCCAGCUACUUGGGAGGCUGAGGCA<br>GGAGAAUGGCGUGAACCCGGGAGGCGGAGCUU<br>GCAGUGAGCCGAGAUCCCGCCACUGCACUCCA<br>GCCUGGGCGACAGAGCGAGACGUCUC<br>AAAAAAAAAAAAAAAAAAAAAAAAA	TPRT assay, Fig. 1C
AluJ SINE 25A (AJ, 306nt)	GGCCGGGCGCGGUGGCUCACGCCUGUAAUCCC<br>AGCACUUUGGGAGGCCGAGGCGGGAGGAUUG<br>CUUGAGCCCAGGAGUUCGAGACCAGCCUGGGC<br>AACAUAGCGAGACCCCGUCUCUACAAAAAUAC<br>AAAAAUUAGCCGGGCGUGGUGGCGCGCGCCUG<br>UAGUCCCAGCUACUCGGGAGGCUGAGGCAGGA<br>GGAUCGCUUGAGCCCAGGAGUUCGAGGCUGCA<br>GUGAGCUAUGAUCGCGCCACUGCACUCCGACC<br>UGGGCGACAGAGCGAGACCCUGUCUC<br>AAAAAAAAAAAAAAAAAAAAAAAAA	TPRT assay, Fig. 1C
AluJ half SINE 25A (AJh, 141nt)	GGCCGGGCGCGGUGGCUCACGCCUGUAAUCCC<br>AGCACUUUGGGAGGCCGAGGCGGGAGGAUUG<br>CUUGAGCCCAGGAGUUCGAGACCAGCCUGGG<br>CAACAUAGCGAGACCCCGUCUC<br>AAAAAAAAAAAAAAAAAAAAAAAAA	TPRT assay, Fig. 1C-D, 2C, 2G, 3D, 3F, 4B-E
L1 3'UTR 25A (L1, 231nt)	GACAAUGAGAUCACAUGGACACAGGAAGGGG<br>AAUAUCACACUCUGGGGACUGUGGUGGGGU<br>CGGGGGAGGGGGGAGGGAUAGCAUUGGGAG<br>AUAUACCUAAUGCUAGAUGACACAUUAGUG<br>GGUGCAGCGCACCAGCAUGGCACAUGUAUA<br>CAUAUGUAACUAACCUGCACAAUGUGCACA<br>UGUACCCUAAAACUUAGAGUAUAAU<br>AAAAAAAAAAAAAAAAAAAAAAAAA	TPRT assay, Fig. 1C
L1 3'UTR ΔG-quadruplex 25A (L1Δ, 149nt)	GAUAGCAUUGGGAGAUAUACCUAAUGCUAGA<br>UGACACAUUAGUGGGUGCAGCGCACCAGCAU<br>GGCACAUGUAUACAUAUGUAACUAACCUGCA<br>CAAUGUGCACAUGUACCCUAAAACUUAGAGU<br>AUAAUAAAAAAAAAAAAAAAAAAAA	TPRT assay, Fig. 1C
AluJ half SINE EM (AJh-EM, 141nt)	GGCCGGGCGCGGUGGCUCACGCCUGUAAUCCC<br>AGCACUUUGGGAGGCCGAGGCGGGAGGAUUG<br>CUUGAGCCCAGGAGUUCGAGACCAGCCUGGG<br>CAACAUAGCGAGACCCCGUCUC<br>AAAAAAAAAAAAAAAAAAAAAAAGC	Cryo-EM structure, Fig. 1E
Synthetic template RNA (74nt)	GCAGACUGCGUAGGCGUGCAGGAACCUGCACG<br>CCUACGCAGUCUGCAAAAAAAAAAAAAAAACAA<br>UAUCGGCGCG	Cryo-EM structure, Fig. 1F
AluJ half SINE 75A (AJh 75A, 191nt)	GGCCGGGCGCGGUGGCUCACGCCUGUAAUCCC<br>AGCACUUUGGGAGGCCGAGGCGGGAGGAUUG<br>CUUGAGCCCAGGAGUUCGAGACCAGCCUGGG<br>CAACAUAGCGAGACCCCGUCUC<br>AAAAAAAAAAAAAAAAAAAAAAAAA<br>AAAAAAAAAAAAAAAAAAAAAAAAA	TPRT assay, Fig. 2C

	AAAAAAAAAAAAAAAAAAAAAAAAA	
AluJ half SINE 50A (AJh 50A, 166nt)	GGCCGGGCGCGGUGGCUCACGCCUGUAAUCCC AGCACUUUGGGAGGCCGAGGCGGGAGGAUUG CUUGAGCCCAGGAGUUCGAGACCAGCCUGGG CAACAUAGCGAGACCCCGUCUC AAAAAAAAAAAAAAAAAAAAAAAAA AAAAAAAAAAAAAAAAAAAAAAAAA	TPRT assay, Fig. 2C
AluJ half SINE 20A (AJh 20A, 136nt)	GGCCGGGCGCGGUGGCUCACGCCUGUAAUCCC AGCACUUUGGGAGGCCGAGGCGGGAGGAUUG CUUGAGCCCAGGAGUUCGAGACCAGCCUGGG CAACAUAGCGAGACCCCGUCUC AAAAAAAAAAAAAAAAAAAA	TPRT assay, Fig. 2C
AluJ half SINE 15A (AJh 15A, 131nt)	GGCCGGGCGCGGUGGCUCACGCCUGUAAUCCC AGCACUUUGGGAGGCCGAGGCGGGAGGAUUG CUUGAGCCCAGGAGUUCGAGACCAGCCUGGG CAACAUAGCGAGACCCCGUCUC AAAAAAAAAAAAAAA	TPRT assay, Fig. 2C
AluJ half SINE 10A (AJh 10A, 126nt)	GGCCGGGCGCGGUGGCUCACGCCUGUAAUCCC AGCACUUUGGGAGGCCGAGGCGGGAGGAUUG CUUGAGCCCAGGAGUUCGAGACCAGCCUGGG CAACAUAGCGAGACCCCGUCUC AAAAAAAAAA	TPRT assay, Fig. 2C
AluJ half SINE 5A (AJh 5A, 121nt)	GGCCGGGCGCGGUGGCUCACGCCUGUAAUCCC AGCACUUUGGGAGGCCGAGGCGGGAGGAUUG CUUGAGCCCAGGAGUUCGAGACCAGCCUGGG CAACAUAGCGAGACCCCGUCUCAAAAA	TPRT assay, Fig. 2C
AluJ half SINE 25N (AJh 25N, 141nt)	GGCCGGGCGCGGUGGCUCACGCCUGUAAUCCCA GCACUUUGGGAGGCCGAGGCGGGAGGAUUGCUU GAGCCCAGGAGUUCGAGACCAGCCUGGGCAACA UAGCGAGACCCCGUCUCGGUAACGAGAACUGUC AUGCACCCC	TPRT assay, Fig. 2C
AluJ half SINE 20N 5A (AJh 20N5A, 141nt)	GGCCGGGCGCGGUGGCUCACGCCUGUAAUCCCA GCACUUUGGGAGGCCGAGGCGGGAGGAUUGCUU GAGCCCAGGAGUUCGAGACCAGCCUGGGCAACA UAGCGAGACCCCGUCUCGGUAACGAGAACUGUC AUGCAAAAA	TPRT assay, Fig. 2C
AluJ half SINE modified 25A (AJhm, 142nt)	GGCCGGGCGCGGUGGCUCACGCCUGUAAUCCCA GCACUUUGGGAGGCCGAGGCGGGAGGAUUGCUU GAGCCCAGGAGUUCGAGACCAGCCUGGGCAACA UAGCGAUCCUCCCGUCUC AAAAAAAAAAAAAAAAAAAAAAAAA	TPRT assay, Fig. 3F
AluJ half SINE unfolded 25A (AJh-uf, 141nt)	GGCCGGGCGCGGUGGCUCACGCCUGUAAUCCCA GCACUUUGGGAGGCCGAGGCGGGAGGAUUGCUU GAGCCCAGGAGUUCGAGACCAGCCUGGGCAACA UAACUAGACUGACAGAG AAAAAAAAAAAAAAAAAAAAAAAAA	TPRT assay, Fig. 3F
RT template RNA (129nt)	GGUAAUAGAACUGUCAUUCAACCCCAAAAAUGAAGU AAUGGGUAAUAGAACUGUCAUUCAACCCCAAAAAUC UAGUAAUGGGUAAUAGAACUGUCAUUCAACCCCAAA AAUCUAGUAAUGCGCGACUGC	RT assay, ED Fig. 2
**DNA**		
Target site (7bp 5' of nick site, 9nt T-rich 5' overhang)	GCTTTTTTTCCTTTTTAAGGACGCATGATGCGGAAACA ATGCATCAC (top strand)	TPRT assay, Fig. 1C-D, 2C, 2G, 3D, 3F, 4B-E

	GTGATGCATTGTTTCCGCATCATGCGTCCTTAAAAAGG (bottom strand)	
Target site (5bp 5' of nick site, 9nt T-rich 5' overhang)	GCTTTTTTTTTTTTAACCGGACGCATGATGCGGAAACAATGCATCAC (top strand) GTGATGCATTGTTTCCGCATCATGCGTCCGGTTAAAAA (bottom strand)	TPRT assay, Fig. 4C, Extended Data Fig. 7-8
Target site (9bp 5' of nick site, 9nt T-rich 5' overhang)	GCTTTTTTTCGGCTTTTTAAACGCATGATGCGGAAACAATGCATCAC (top strand) GTGATGCATTGTTTCCGCATCATGCGTTTAAAAAGCCG (bottom strand)	TPRT assay, Fig. 4C, Extended Data Fig. 7-8
Target site (11bp 5' of nick site, 9nt T-rich 5' overhang)	GCTTTTTTTCGGACCTTTTTAAGCATGATGCGGAAACAATGCATCAC (top strand) GTGATGCATTGTTTCCGCATCATGCTTAAAAAGGTCCG (bottom strand)	TPRT assay, Fig. 4C, C, Extended Data Fig. 7-8
Target site (13bp 5' of nick site, 9nt T-rich 5' overhang)	GCTTTTTTTCGGACGCCTTTTTAAATGATGCGGAAACAATGCATCAC (top strand) GTGATGCATTGTTTCCGCATCATGCTTAAAAAGGTCCG (bottom strand)	TPRT assay, Fig. 4C, C, Extended Data Fig. 7-8
Target site (18bp 5' of nick site, 9nt T-rich 5' overhang)	GCTTTTTTTCGGACGCATGATCTTTTTAAGCGGAAACAATGCATCAC (top strand) GTGATGCATTGTTTCCGCTTAAAAAGATCATGCGTCCG (bottom strand)	TPRT assay, Fig. 4B, Extended Data Fig. 7-8
Target site (26bp 5' of nick site, 9nt T-rich 5' overhang)	GCTTTTTTTCGGACGCATGATGCGGAAACCTTTTTAAAATGCATCAC (top strand) GTGATGCATTTTAAAAAGGTTTCCGCATCATGCGTCCG (bottom strand)	TPRT assay, Fig. 4B, Extended Data Fig. 7-8
Target site (7bp 5' of nick site, 0nt 5' overhang)	CCTTTTTAAGGACGCATGATGCGGAAACAATGCATCAC (top strand) GTGATGCATTGTTTCCGCATCATGCGTCCTTAAAAAGG (bottom strand)	TPRT assay, Fig. 4D
Target site (7bp 5' of nick site, 9nt 5' overhang)	CGCGCCGAACCTTTTTAAGGACGCATGATGCGGAAACAATGCATCAC (top strand) GTGATGCATTGTTTCCGCATCATGCGTCCTTAAAAAGG (bottom strand)	TPRT assay, Fig. 4D
Target site (7bp 5' of nick site, 9nt 5' overhang with alternative T-rich sequence lanes)	GCAGTTTTTCCTTTTTAAGGACGCATGATGCGGAAACAATGCATCAC (top strand) GTGATGCATTGTTTCCGCATCATGCGTCCTTAAAAAGG (bottom strand)	TPRT assay, Fig. 4D final 3 lanes in panel
Target site (7bp 5' of nick site, 17nt 5' overhang)	CGCGCCGAGCTTTTTTTCCTTTTTAAGGACGCATGATGCGGAAACAATGCATCAC (top strand) GTGATGCATTGTTTCCGCATCATGCGTCCTTAAAAAGG (bottom strand)	TPRT assay, Fig. 4E
Target site (7bp 5' of nick site, 27nt 5' overhang)	ACGTCTCTATCGCGCCGAGCTTTTTTTCCTTTTTAAGGACGCATGATGCGGAAACAATGCATCAC (top strand) GTGATGCATTGTTTCCGCATCATGCGTCCTTAAAAAGG (bottom strand)	TPRT assay, Fig. 4E
9nt RT assay primer	GCAGTCGCG	RT assay, ED Fig. 2
7nt EM primer for SINE RNA	GCTTTTT	Cryo-EM structure, Fig. 1E
8nt EM primer for synthetic template RNA	CGCGCCGA	Cryo-EM structure, Fig. 1F
