# Supplementary material for: Template and target-site recognition by human LINE-1 in retrotransposition
Source: Nature. 2023 Dec 14;626(7997):186–93. doi: 10.1038/s41586-023-06933-5 (PMC10830416; doi:10.1038/s41586-023-06933-5)
Supplement: Supplementary file 2 — Reporting Summary [file 41586_2023_6933_MOESM2_ESM.pdf]

nature portfolio

Corresponding author(s): Eva Nogales, Kathleen Collins

Last updated by author(s): Nov 7, 2023

Reporting Summary

Nature Portfolio wishes to improve the reproducibility of the work that we publish. This form provides structure for consistency and transparency in reporting. For further information on Nature Portfolio policies, see our Editorial Policies and the Editorial Policy Checklist.

Statistics

For all statistical analyses, confirm that the following items are present in the figure legend, table legend, main text, or Methods section.

n/a	Confirmed	
☐	☒	The exact sample size (*n*) for each experimental group/condition, given as a discrete number and unit of measurement
☐	☒	A statement on whether measurements were taken from distinct samples or whether the same sample was measured repeatedly
☒	☐	The statistical test(s) used AND whether they are one- or two-sided<br>*Only common tests should be described solely by name; describe more complex techniques in the Methods section.*
☒	☐	A description of all covariates tested
☒	☐	A description of any assumptions or corrections, such as tests of normality and adjustment for multiple comparisons
☐	☒	A full description of the statistical parameters including central tendency (e.g. means) or other basic estimates (e.g. regression coefficient) AND variation (e.g. standard deviation) or associated estimates of uncertainty (e.g. confidence intervals)
☒	☐	For null hypothesis testing, the test statistic (e.g. *F*, *t*, *r*) with confidence intervals, effect sizes, degrees of freedom and *P* value noted<br>*Give P values as exact values whenever suitable.*
☒	☐	For Bayesian analysis, information on the choice of priors and Markov chain Monte Carlo settings
☒	☐	For hierarchical and complex designs, identification of the appropriate level for tests and full reporting of outcomes
☒	☐	Estimates of effect sizes (e.g. Cohen's *d*, Pearson's *r*), indicating how they were calculated

Our web collection on statistics for biologists contains articles on many of the points above.

Software and code

Policy information about availability of computer code

Data collection	Serial EM 4-0-20 for cryo-EM data collection
Data analysis	The EM softwares used: Relion 3.1.1, cryoSPARC v.3, cryoSPARC v.4, Cryolo 1.7.6. Structures were built using Coot 0.8.9, Chimera 1.14, ChimeraX 1.3, Phenix 1.20, Pymol 2.5.4. Gels were analyzed using ImageJ (Fiji) 2.1.0

For manuscripts utilizing custom algorithms or software that are central to the research but not yet described in published literature, software must be made available to editors and reviewers. We strongly encourage code deposition in a community repository (e.g. GitHub). See the Nature Portfolio guidelines for submitting code & software for further information.

Data

Policy information about availability of data

All manuscripts must include a data availability statement. This statement should provide the following information, where applicable:

- Accession codes, unique identifiers, or web links for publicly available datasets
- A description of any restrictions on data availability
- For clinical datasets or third party data, please ensure that the statement adheres to our policy

The described cryo-EM maps and coordinate files were deposited in the Electron Microscopy Data Bank (EMDB) with accession code EMD-42637 and in Protein Data Bank (PDB) with accession code PDB 8UW3. All other datasets, reagents or resources generated during this study are available upon request from the corresponding authors.

Research involving human participants, their data, or biological material

Policy information about studies with human participants or human data. See also policy information about sex, gender (identity/presentation), and sexual orientation and race, ethnicity and racism.

Reporting on sex and gender	n/a
Reporting on race, ethnicity, or other socially relevant groupings	n/a
Population characteristics	n/a
Recruitment	n/a
Ethics oversight	n/a

Note that full information on the approval of the study protocol must also be provided in the manuscript.

Field-specific reporting

Please select the one below that is the best fit for your research. If you are not sure, read the appropriate sections before making your selection.

☒ Life sciences ☐ Behavioural & social sciences ☐ Ecological, evolutionary & environmental sciences

For a reference copy of the document with all sections, see nature.com/documents/nr-reporting-summary-flat.pdf

Life sciences study design

All studies must disclose on these points even when the disclosure is negative.

Sample size	In total 23,874 microscope raw movies collected from two different grid preparations were used for data processing of the highest resolution structure, sufficient to provide a high resolution structure. This data size was determined in order to reconstruct a high-resolution cryo-EM map for structure determination were obtained (at around 3 angstrom resolution). For low resolution, Alu structure 23,878 microscope raw movies collected from one grid preparations were used for data processing to yield a structure where protein and RNA densities could be clearly fitted at around 4 angstrom resolution. For biochemical assays, at least three independent biological replicates were performed, as recommended and as is the standard in similar works.
Data exclusions	Poor resolution data was excluded from cryo-EM analysis through 2D classifications and 3D classifications. This is standard step in single-particle cryo-EM analysis workflow and necessary to obtain highest resolution structures.
Replication	All biochemical experiments were repeated in three or more independent replicates, specified within the figure legends for individual experiments. All replicates which showed similar results. Data from all replicates were pooled for quantification and reported in bar graphs
Randomization	In the Fourier shell correlation (FSC) measurement step of the Relion 3.1 data processing pipeline, data were randomly divided into two halves resulting in two independently determined 3D volumes that were used for the FSC calculation.
Blinding	Data division in the FSC calculation step is a computer-based, unbiased process. Individual processing of different datasets collected from different human heart samples gave rise to the same 3D structures.

Reporting for specific materials, systems and methods

We require information from authors about some types of materials, experimental systems and methods used in many studies. Here, indicate whether each material, system or method listed is relevant to your study. If you are not sure if a list item applies to your research, read the appropriate section before selecting a response.

Materials & experimental systems

n/a	Involved in the study	
☒	☐	Antibodies
☐	☒	Eukaryotic cell lines
☒	☐	Palaeontology and archaeology
☒	☐	Animals and other organisms
☒	☐	Clinical data
☒	☐	Dual use research of concern
☒	☐	Plants

Methods

n/a	Involved in the study	
☒	☐	ChIP-seq
☒	☐	Flow cytometry
☒	☐	MRI-based neuroimaging

Eukaryotic cell lines

Policy information about cell lines and Sex and Gender in Research

Cell line source(s)	SF9 cell line for baculovirus generation. SF9 and High5 cells lines for protein production. The SF9 and High5 cells were obtained from Invitrogen, ThermoFisher.
Authentication	No authentication of cell lines was performed as they were purchased from reliable commercial sources.
Mycoplasma contamination	Cells were tested for mycoplasma contamination and were found to be negative. Cell lines were monitored for doubling time and correct morphology.
Commonly misidentified lines (See ICLAC register)	No misidentified cell lines were used in this work.

Plants

Seed stocks	n/a
Novel plant genotypes	n/a
Authentication	n/a
